# Canakinumab and mycophenolate mofetil in managing proteinuria/renal amyloidosis secondary to adult-onset Still’s disease

**DOI:** 10.1093/rap/rkad046

**Published:** 2023-05-02

**Authors:** Jin Feng, Lea Meir, Olivia Ghaw

**Affiliations:** Department of Medicine, Icahn School of Medicine at Mount Sinai, Mount Sinai Morningside/West, New York, NY, USA; Division of Rheumatology, Department of Medicine, Icahn School at Mount Sinai, Mount Sinai Hospital, New York, NY, USA; Division of Rheumatology, Department of Medicine, Icahn School at Mount Sinai, Mount Sinai Hospital, New York, NY, USA

Key message

- Canakinumab and MMF may have roles in decreasing proteinuria secondary to renal amyloidosis.

Dear Editor, Renal amyloidosis is a rare complication of Adult-onset Still’s disease. We report a case of proteinuria secondary to renal amyloidosis, managed with canakinumab and MMF, as shown in Fig. 1.

**Figure 1.**
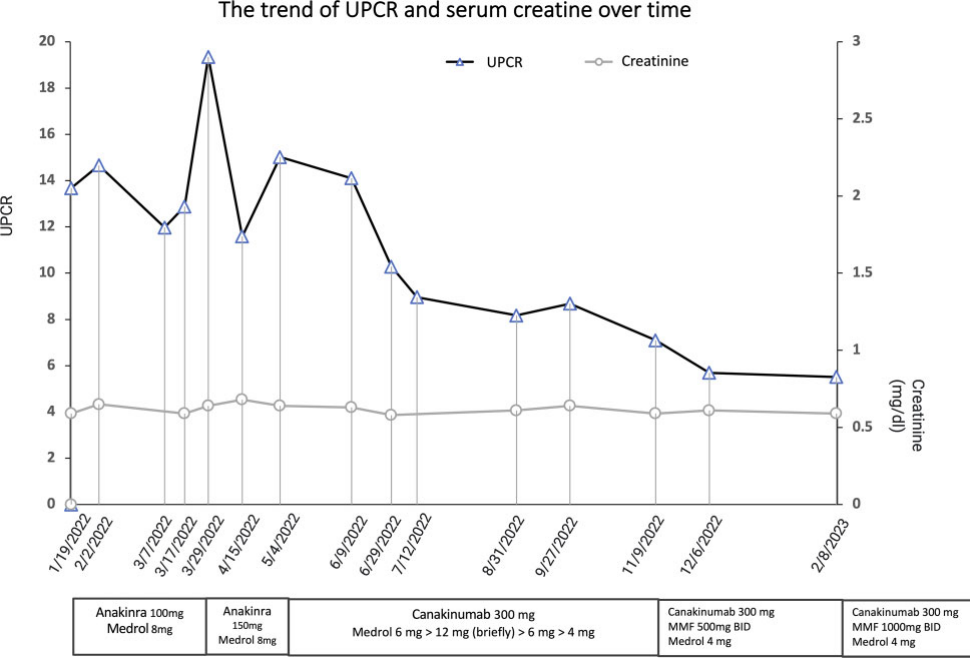
The UPCR gradually improved while the treatment switched to canakinumab and MMF

A 58-year-old female with a past medical history of GERD, iron-deficiency anaemia and Adult-onset Still’s disease (AOSD; diagnosed in 2020), complicated with erosive arthritis, presented to the clinic to establish care in September 2021 following her hospitalization due to AOSD flaring. She was last treated with colchicine and upadacitinib but self-discontinued in July due to gastrointestinal side effects. She subsequently flared and was hospitalized in September with symptoms of diffuse maculopapular rash, polyarticular arthralgias and intermittent high fevers since off treatment for 1 month. During the hospitalization, she was evaluated by rheumatology and was discharged on anakinra 100 mg/day and methylprednisolone taper. Previously at an outside clinic she was treated with multiple agents, including NSAIDs, methylprednisolone (Medrol), methotrexate (stopped due to intractable vomiting), infliximab (stopped due to allergic reaction) and adalimumab (stopped due to inefficacy).

With this regimen, the patient’s inflammatory markers improved and her symptoms stabilized, except for worsening lower extremity oedema, which was initially attributed to steroid-based fluid retention. However, urinalysis showed significant proteinuria with a urine protein:creatinine ratio (UPCR) of 13.68 in January 2022. Renal biopsy was done 2 days later, showing AA subtype of renal amyloidosis in mesangial regions and vessel walls. A diagnosis of renal amyloidosis secondary to poorly controlled AOSD was made. The patient was clinically quiescent since she had no synovitis, fevers or rash, so methylprednisolone was further tapered down to 4 mg. In March 2022, given that her proteinuria was worsening with a maximum UPCR of 19.35, her anakinra was increased to 150 mg/day. However, her proteinuria was refractory to the anakinra dosage increase, so 2 months later her regimen was switched to canakinumab 300 mg every month. Her UPCR gradually trended downwards beginning in June, from 14.1 to 7.1, with improving pedal oedema and foamy urine. The patient’s AOSD otherwise remained clinically quiet. Four months later, in November, MMF 500 mg twice a day was added to the patient’s regimen because the rate of her UPCR decline had begun to plateau (UPCR remained at 7–8) and due to reported variable swelling and pain in her knees and hips. In February 2023, approximately 4 months later, her proteinuria has much improved, with a 19.85% decrease in her UPCR from 7.10 to 5.69 while on canakinumab 300 mg monthly, MMF 500 mg twice a day and methylprednisolone 4 mg/day. Even though her proteinuria has improved drastically since a year ago from a UPCR maximum of 19.35 down to 5.5, it has remained above the nephrotic range. Thus MMF was increased to 1 g twice a day while continuing canakinumab and methylprednisolone. She will continue to follow up with rheumatology and nephrology to monitor her progress. Her creatinine has remained normal throughout the visits. Given that she received monthly IV iron from February to August 2022, her ferritin levels did not reflect her AOSD activity. The patient never underwent genetic testing because she has a classic AOSD presentation and denies related family history.

AOSD is a rare autoinflammatory disorder with an approximate prevalence of 1–34 cases per million persons [1]. Renal AA amyloidosis, one of AOSD’s complications, is even rarer, with an estimated prevalence of 4.7–14.3%. Renal amyloidosis usually manifests 18 months–30 years after diagnosis, with the most common initial presentation being proteinuria and nephrotic syndrome [2]. Previous studies show that proinflammatory cytokines such as IL-1β, IL-6, IL-8, IL-18 and TNF-α are crucial in AOSD pathogenesis [3]. Such findings corroborate the efficacy of biologics and their important roles in AOSD in addition to traditional regimens of DMARDs, NSAIDs and steroids [4]. Several case reports have described promising results of renal AA amyloidosis treated with TNF inhibitors, IL-6 antagonists and IL-1 antagonists [1, 5, 6]. Canakinumab caused a significant decrease in the UPCR from 15.02 to 5.51 in this case. This will be an addition to the current literature, which is still scarce given the rarity of this condition. MMF, an uncommon intervention in AOSD, was chosen as an add-on therapy for this patient with a further decrease in UPCR. Since her proteinuria remains in the nephrotic range while on canakinumab, MMF may bring benefits as some studies have reported potential efficacy in AOSD [7].

Although there has been established proof regarding canakinumab’s efficacy in managing AOSD, there is so far limited data on its role in reducing proteinuria secondary to renal AA amyloidosis of AOSD [8]. This case highlights the potential role of canakinumab as another potent biologic to be used in the treatment of renal amyloidosis. It also demonstrates a substantial decrease in proteinuria with MMF as an add-on therapy. More studies need to be performed to support our findings.

## Data Availability

The data underlying this article cannot be shared publicly for the privacy of the individual who participated in the case study. The data will be shared upon reasonable request to the corresponding author.
